# Distributed slow-wave dynamics during sleep predict memory consolidation and its impairment in schizophrenia

**DOI:** 10.1038/s41537-019-0086-8

**Published:** 2019-11-04

**Authors:** Ullrich Bartsch, Andrew J. Simpkin, Charmaine Demanuele, Erin Wamsley, Hugh M. Marston, Matthew W. Jones

**Affiliations:** 1 Translational & Integrative Neuroscience, Lilly Research Centre, Windlesham, Surrey GU20 6PH UK; 2 School of Physiology, Pharmacology & Neuroscience, University of Bristol, Biomedical Sciences Building, University Walk, Bristol, BS8 1TD UK; 3 School of Mathematics, Statistics and Applied Mathematics, National University of Ireland, Galway, H91 TK33 Ireland; 4 Department of Psychiatry, Massachusetts General Hospital, Charlestown, MA 02215 USA; 5 Athinoula A. Martinos Centicaer for Biomedl Imaging, Charlestown, MA 02129 USA; 6 Harvard Medical School, Boston, MA 02115 USA; 7 Department of Psychology, Furman University, Greenville, SC 29613 USA; 8 Present Address: Early Clinical Development, Pfizer Inc., Cambridge, MA USA

**Keywords:** Schizophrenia, Learning and memory

## Abstract

The slow waves (SW) of non-rapid eye movement (NREM) sleep reflect neocortical components of network activity during sleep-dependent information processing; their disruption may therefore impair memory consolidation. Here, we quantify sleep-dependent consolidation of motor sequence memory, alongside sleep EEG-derived SW properties and synchronisation, and SW–spindle coupling in 21 patients suffering from schizophrenia and 19 healthy volunteers. Impaired memory consolidation in patients culminated in an overnight improvement in motor sequence task performance of only 1.6%, compared with 15% in controls. During sleep after learning, SW amplitudes and densities were comparable in healthy controls and patients. However, healthy controls showed a significant 45% increase in frontal-to-occipital SW coherence during sleep after motor learning in comparison with a baseline night (baseline: 0.22 ± 0.05, learning: 0.32 ± 0.05); patient EEG failed to show this increase (baseline: 0.22 ± 0.04, learning: 0.19 ± 0.04). The experience-dependent nesting of spindles in SW was similarly disrupted in patients: frontal-to-occipital SW–spindle phase-amplitude coupling (PAC) significantly increased after learning in healthy controls (modulation index baseline: 0.17 ± 0.02, learning: 0.22 ± 0.02) but not in patients (baseline: 0.13 ± 0.02, learning: 0.14 ± 0.02). Partial least-squares regression modelling of coherence and PAC data from all electrode pairs confirmed distributed SW coherence and SW–spindle coordination as superior predictors of overnight memory consolidation in healthy controls but not in patients. Quantifying the full repertoire of NREM EEG oscillations and their long-range covariance therefore presents learning-dependent changes in distributed SW and spindle coordination as fingerprints of impaired cognition in schizophrenia.

## Introduction

NREM sleep EEG is dominated by low-frequency oscillations, which co-occur in systematically varying proportions during the night: deep NREM sleep (stage N3, or “slow wave sleep”) features pronounced 0.5–4 Hz power (sometimes subdivided into SW at 0.5–1.5 Hz and delta oscillations at 1.5–4 Hz), whereas lighter NREM (stage N2) encompasses the majority of 12–15 Hz spindle oscillations.

SWs and spindles are cortical signatures of the patterned thalamocortical and limbic network activity that supports NREM sleep’s roles in overnight memory consolidation.^1–4^ For example, regional increases in SW density and amplitude correlate with declarative memory consolidation,^5^ and augmenting SW activity during sleep can enhance memory.^6–8^ The duration of stage N2 sleep and spindle density also correlates with performance in both procedural and declarative memory tasks.^9–13^

It follows that memory impairments in disease may be caused or exacerbated by disrupted NREM neurophysiology. This link is best characterised in schizophrenia,^14^ where deficits in sleep-dependent memory consolidation correlate with aberrant EEG signatures during NREM sleep.^15–17^ Some studies report that deep NREM sleep is reduced in patients,^18–20^ and attenuated SW power^21–23^ has also been linked to cognitive deficits.^17,24,25^ However, not all studies report SW abnormalities.^15^ Schizophrenia has more consistently been associated with reductions in spindle density or sigma power in both patients and first-degree relatives,^15,16,26–28^ with spindle measures showing some correlation with impaired sleep-dependent memory consolidation in patients.^29^

Paralleling the emergence of this clinical evidence linking disrupted sleep-dependent neural network activity with impaired memory consolidation, deep-brain recordings in rodents have detailed the roles of NREM network oscillations in shaping the patterned neural activity underpinning mnemonic processing. Much of this rodent work has focused on the hippocampus, where pyramidal neuron-spiking patterns during NREM “replay” sequential activity encoding recent behavioural experience.^30^ However, the timing of hippocampal activity during NREM is influenced by SW activity^31–33^ and is in turn coordinated with spindles in the neocortex.^34–38^ Beyond examining SW or spindles per se, quantifying the coordination between ripples, spindles and SW can therefore illuminate the mechanisms and functions of brain activity during sleep in health and disease.^39,40^

Philips et al.^41^ began to address disordered interdependence of SW, spindles and ripples by using the Methylazoxymethanol acetate (MAM)–E17 rat neurodevelopmental model of schizophrenia,^42^ demonstrating that the MAM–E17 model harbours reduced NREM SW power and reduced coherence of SW between frontal and occipital cortices. This reduced SW coherence was accompanied by impaired frontal slow-wave phase coupling to posterior cortical spindle amplitude and decoupling of cortical spindle and hippocampal ripple oscillations, potentially as a consequence of interneuronal dysfunction.^43^ Consistent with predictions from this rodent work, a recent analysis by Demanuele et al. showed that regression models incorporating both spindle density and a measure of spindle–SW phase-locking were able to predict memory performance in schizophrenia patients.^44^ However, Demanuele et al. focused on local SW–spindle phase-locking (SW and spindles detected on the same EEG recording site), which did not predict sleep-dependent memory in healthy participants.

These results prompted us to investigate properties of SWs and their interrelationship with spindles across different cortical sites by using the same sleep EEG recordings from chronic medicated schizophrenia patients and demographically matched control participants. Wamsley et al. reported reduced spindle density and 12–13.5 Hz (low sigma) power during N2 sleep in these patients, alongside reduced spindle coherence between centro-occipital EEG electrodes. Of these measures, spindle densities correlated with overnight improvement on the finger-tapping motor sequence task (MST),^10,45^ but only in schizophrenia patients and not in healthy volunteers. Here, we show that quantification of SW properties, SW coherence and SW–spindle phase-amplitude coupling across the cortical mantle strengthens and clarifies links between NREM neurophysiology and memory consolidation. Our results highlight the importance of long-range network connectivity during NREM as central to mnemonic processing in healthy volunteers, and dissociation of SW coherence from experience in patients diagnosed with schizophrenia.

## Results

### Impaired sleep-dependent motor memory consolidation in SCZ

**Fig. 1:**
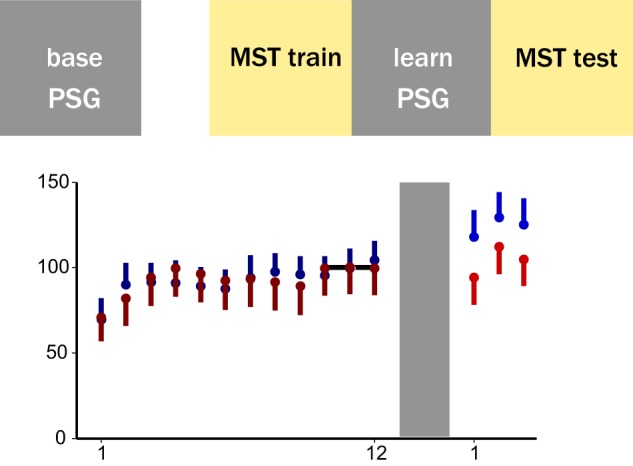
Patients with schizophrenia show impaired sleep-dependent memory consolidation in the motor sequence task (MST). **a** Timeline of behavioural testing and polysomnography. The baseline night 1 (base) was not preceded by any behavioural training. The following night, participants were trained on the MST 1 h before their regular bedtime (MST train) and were tested the following morning, after up to 10 h of sleep during night 2 (learn). **b** MST performance. Behavioural results for all participants included in night 2 EEG analyses (healthy controls, CON, *n* = 15; patients, SCZ, *n* = 21). The panel shows the mean of correct sequences per 30-s epoch with the standard error indicated as one-sided error bar. Because SCZ patients typed fewer correct sequences during training, task performance (number of correct sequences/30 s) was normalised by the asymptotic level achieved during MST training (mean of last 3 trials, <CON_10–12_ > = 19.05, <SCZ_10–12_ > = 11.52). Controls show a significant improvement on the MST on the next day, whereas SCZ patients do not (improvement in percentage, two-sided *t* test, *p* = 0.025, *t* = 2.3,4 and df: 33.17, see Wamsley et al., 2012, for statistics on a larger sample)

We present novel analyses of a previously acquired dataset.^29^ Briefly, participants were invited to the sleep lab for a baseline (“base”) polysomnography (PSG) recording on night 1. Night 2, which we refer to as the learning night (“learn”, Fig. 1a), was flanked by a motor-sequencing task before (MST train) and after (MST test) sleep. For the current analysis of behaviour, we only included participants with quality-controlled EEG recordings on night 2 (see the “Methods” section). Patients diagnosed with schizophrenia (SCZ) and healthy controls (CON) showed similar normalised learning curves during training on the MST. However, patients did not show overnight improvement on the MST compared with healthy controls (improvement percentages CON: mean 15.93%, SD = 13.68%; SCZ: mean 1.62%, SD = 22.88%; two-sided *t* test, *p* = 0.025; t-stat: 2.34; df: 33.17, Fig. 1b, see Wamsley et al.^29^ for results from a larger sample).

### Conserved slow-wave event properties in SCZ

**Fig. 2:**
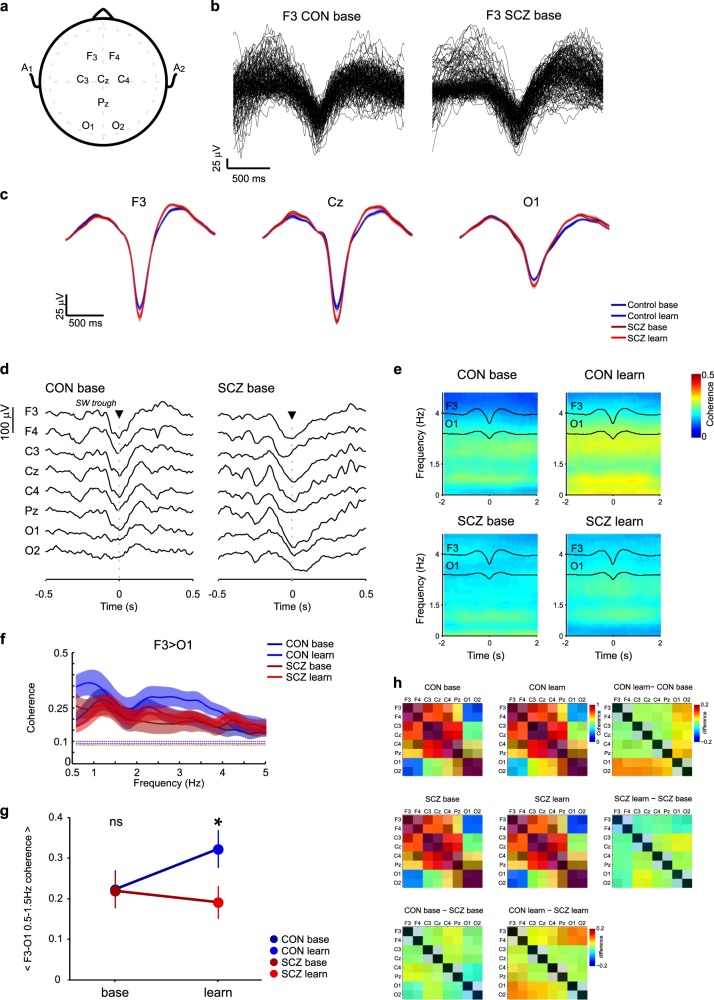
Normal SW amplitudes but reduced learning-dependent SW coherence in SCZ during NREM sleep. **a** EEG recording configuration. EEG recording positions diagram as defined by the 10–20 system. **b** Example SW. Overlay plot of all automatically detected raw EEG SW from one healthy control (CON) and one patient (SCZ) during baseline NREM sleep at electrode F3. **c** Wave- triggered averages of detected SW at F3, Cz and O1. Wave-triggered SW averages in controls (dark blue: baseline, light blue: learning, error areas show standard error of mean, SEM) and patients (dark red: baseline, light red: learning, error areas show standard error of mean). **d** Example EEG traces during SW at F3 in CON and SCZ. Example of an automatically detected SW at F3 and the corresponding 1-s window of simultaneously recorded activity across all other electrodes. **e** Average SW-triggered multi-tapered coherograms between F3 and O1 during NREM sleep. All trough times from SWs detected at F3 were used to select a ± 2-s window at F3 and O1 to compute group- and night-averaged sliding-window multi-taper coherograms (included F3–O1 pairs: *N*_CON_ = 11, *N*_SCZ_ = 14). Wave-triggered averages of SWs at the trigger channel (F3, top trace) and target channel (O1, bottom trace) are overlaid. **f** Average SW-triggered coherency during NREM sleep. All peak times from SWs detected at F3 were used to compute the group- and night-averaged coherency between F3 and O1 in a ± 2-s window (included F3–O1 pairs: *N*_CON_ = 11, *N*_SCZ_ = 14) including jackknife 95% intervals (shaded error areas). **g** Interaction plot for average SW-triggered slow (0.5–1.5 Hz) coherence. Least-squares estimates of the group means with 95% confidence intervals from a linear mixed-model analysis of 0.5–1.5 Hz coherence values (*p < 0.05; see supplementary information M2 for mixed-model statistics). **h** Brain-wide differences in SW-triggered slow coherence. Average coherence values (0.5–1.5 Hz, ± 2 s around SW) during baseline and learning in each group were plotted in coherence matrices set by electrode location on the scalp. Night and group differences are shown next to average values (as labelled)

We analyzed artefact-free EEG traces from all epochs of N2 and N3 sleep (referred to collectively as NREM sleep) at the electrode positions depicted in Fig. 2a. Comparisons of the basic spectral properties of all NREM sleep epochs show that the topographic distributions of SW and spindle power were similar in volunteers and patients (Supplementary Fig. S1a, b), though spectra at individual electrodes showed increased power at sub-10-Hz frequencies in patients, particularly over central and occipital electrodes (Supplementary Fig. S1c).

Next, we detected individual SW events on all EEG channels as described by Phillips et al.^43^ (see also Supplementary Fig. S2a), capturing both N2 “K-complexes” at 0.5–2 Hz and N3 “delta waves” at 0.5–2 Hz intrinsic frequency. We were intentionally inclusive about the type of slow NREM events we were analyzing, since their underlying biophysical mechanisms are believed to be highly related.^46,47^ Figure 2b shows typical SWs detected at electrode F3 in one healthy participant and one SCZ patient during the baseline night. There were no differences in SW density, oscillation frequency, amplitude or duration between controls and patients (Supplementary Figs S2 and S3). To quantify SW morphology, we calculated wave-triggered averages (triggered by SW trough times) for each electrode, group and night. Figure 2c shows average SW for F3, Cz and O1 electrodes; although SW tended to be of slightly higher amplitude in patients, these differences were not significant (two-sided Wilcoxon rank-sum test with FDR correction, *p*_BH_ < 0.05; see Supplementary Fig. S2b for bin-by-bin analysis).

### Reduced long-range coupling of 0.5–1.5-Hz slow waves in SCZ

SW tends to originate in frontal cortices and can be either local or travelling waves that, in some instances, are coordinated across multiple cortical areas.^48–52^ To quantify coordination of distributed SW activity across EEG electrodes, we used SW troughs as reference timestamps around which to calculate multi-tapered spectral coherence.^41^

Figure 2e shows group-averaged coherograms for volunteers and patients during baseline and post-learning NREM sleep. In all, 0.5–1.5-Hz SW coherence between electrodes F3 and O1 did not differ between CON and SCZ during the baseline night but showed a marked learning-dependent increase in CON (also shown averaged over NREM in Fig. 2f) that was missing in SCZ patients. A linear mixed-model analysis of Fisher z-transformed average coherence values revealed a fixed effect for recording night, although this main effect did not survive type III Analysis of Variance with Satterwaithe approximation for degrees of freedom (*F*_(1,20.63)_ = 1.56, *p* = 0.23). However, a significant night × group interaction for SW coherence was evident (*F*_(1,20.63)_ = 5.04, *p* = 0.036). SW coherence increased significantly following MST training only in the CON group (CON: baseline: 0.222, SE = 0.047, learning: 0.322, SE = 0.047, *p*_(CON base–CON learn)_ = 0.03), and was significantly higher compared with SCZ during the learning night (SCZ: baseline: 0.219, SE + 0.044, learning: 0.191, SE = 0.040), *p*_(SCZ learn–CON learn)_ = 0.04, see also Supplementary Statistical Methods M1.

To visualise the topography of these group- and learning-dependent changes, average SW coherence values across all electrode pairs are shown for each group and night in Fig. 2h. Note that these matrices are not diagonally symmetrical, since the event-based coherence is dependent on the events detected at a trigger electrode (e.g. F3) that will then select simultaneously recorded data at a target electrode (e.g. C3). Reversing the direction and detecting events at C3 to select data at F3 reveals some directionality in the coupling matrices, since F3 and C3 events are not always synchronous. Following MST training, the CON group exhibited increases in fronto-/central-to-occipital coherence (Fig. 2h, CON learn–CON base) that were not evident in patients. Consequently, comparison between CON and patient SW coherence during the learning night (Fig. 2h, CON learn– SCZ learn) highlights deficits in learning-dependent, long-range coordination of SW activity between frontal/central and occipital cortices in SCZ.

Overall, these SW analyses show that SCZ patients exhibit conserved SW densities and amplitudes but an absence of learning-dependent coordination of SW activity across the cortical mantle.

### Reduced learning-dependent coupling of SW and spindles in SCZ

**Fig. 3:**
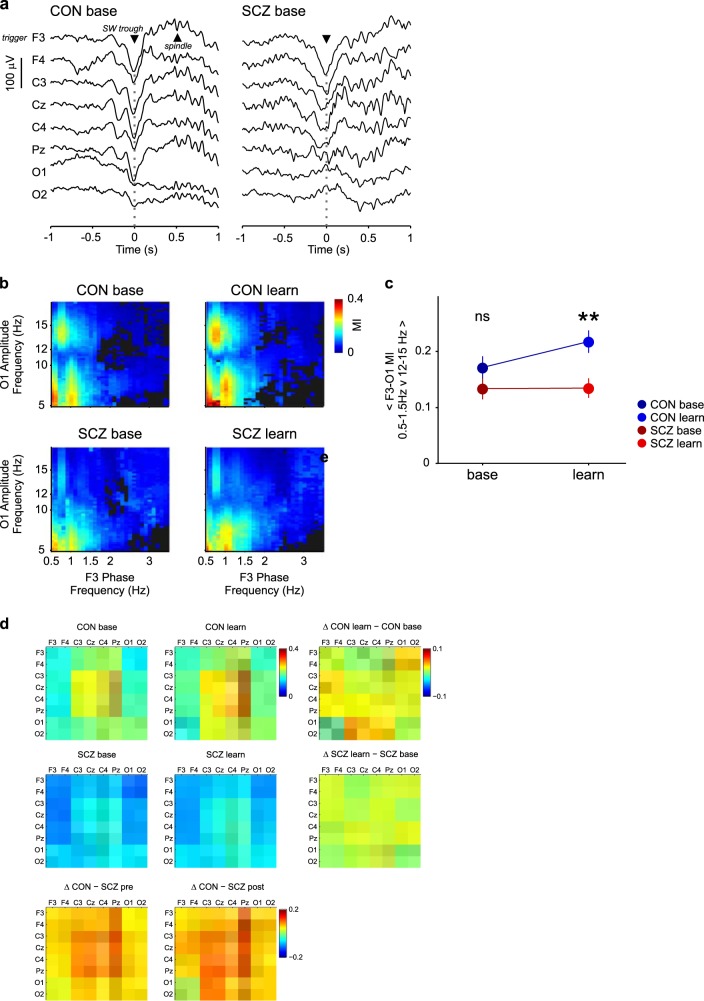
Long-range SW–spindle coupling shows a learning-dependent increase in CON but not in SCZ. **a** SW–spindle co-occurrence during NREM sleep. Examples of a frontally detected SW and subsequent spindle oscillations across multiple channels from one healthy CON individual (CON) and one patient (SCZ) during baseline sleep. **b** SW-triggered frontal–occipital phase-amplitude coupling. The negative peaks of F3-derived SW were used as reference points to calculate a co-modulogram for a ± 2-s window of data at F3 and O1. The F3 trace was used as modulating input (phase) and O1 was used as modulated input (amplitude). Only significant MI values were included in further analysis (n.s. are dark-grey bins, see Methods). The resulting participant co-modulograms were averaged across nights for each group. **c** Interaction plot of mean F3–O1 SW–spindle MI. The mean SW–spindle MI values (0.5–1.5-Hz phase, and 12–15-Hz amplitude) were fed into a linear mixed model. The interaction plot demonstrates the significant interaction effect between night and group in the final model. Least-squares estimation was used to determine group means with 95% confidence intervals from the final model. **d** Brain-wide differences in SW (0.5–1.5 Hz) spindle (12–15 Hz) MI. MI matrices display mean values of SW–spindle PAC and differences between all sensor pairs. Mean MI values (0.5–1.5-Hz phase, and 12–15-Hz amplitude) were plotted against electrode position to create a PAC matrix. Average PAC matrices were calculated for baseline (left) and learning (middle) nights, in each group (CON, top; SCZ, middle), respectively. Differences in SW–spindle PAC between baseline and learning night for both groups were added to the right and below the respective matrices of mean values

The coordination of SW with spindle oscillations has been shown to correlate with memory consolidation^37,53,54^ and can be readily identified in EEG recordings (see examples in Fig. 3a). Demanuele et al.^44^ demonstrated that local slow-wave phase to spindle-amplitude coupling (i.e. derived from signals from the same EEG sensor) can correlate with sleep-dependent memory consolidation, but only in SCZ patients. To test whether distributed, cortex-wide coupling of SW and spindle oscillations is a more sensitive metric of memory consolidation and impairment, we used an established phase-amplitude coupling measure (modulation index, MI)^55,56^ between the most distal electrode locations, F3 and O1. SW frequency-filtered F3 signals were entered as the modulating signal (phase), and target electrode O1 was entered as the modulated signal (amplitude).

Figure 3b shows the resulting average co-modulograms for CON and SCZ groups on both recording nights. Linear mixed-model analysis of the average 0.5–1.5 Hz vs 12–15-Hz MI revealed the main effects for group (Type III analysis with Satterwaithe approximation for degrees of freedom *F*_(1,22.540)_ = 5.78, *p* = 0.025) and recording night (*F*_(1,18.626)_ = 5.30, *p* = 0.033) with a significant group × night interaction (*F*_(1,18.626)_ = 4.72, *p* = 0.043). Post hoc testing confirmed a learning-dependent increase in F3–O1 SW–spindle MI in the CON group (CON MI baseline: 0.171 SE + 0.02, learning: 0.218 and SE = 0.02 *p*_(CON baseline–CON learning)_ = 0.007) that was significantly higher compared with SCZ during the learning night *p*(_SCZ learning–CON learning)_ = 0.004, SCZ MI baseline: 0.133, SE = 0.019 and learning: 0.135. SE = 0.018, Fig. 3c). Consistent with Demanuele et al.,^44^ this effect was not apparent for local (within electrode) SW–spindle PAC, where the MI showed a group effect but no significant interaction between group and night (Supplementary Statistical Methods M4).

Since the interrelationships between SW and spindle timing across different cortical regions proved more sensitive to learning than intra-regional SW–spindle coupling, we calculated SW–spindle PAC for all electrode pairs. In all, 0.5–1.5-Hz SW phase at one electrode (modulating phase) was used to calculate the modulation index of 12–15-Hz spindle power (modulated amplitude) at each electrode; the resulting MI matrices are shown in Fig. 3d. In CON, the most prominent SW–spindle coupling during pre-learning NREM sleep was between Pz and frontal–central electrodes; this coupling increased after learning, particularly for Pz–F4 and more posterior, occipital electrode pairs. Overall, SW–spindle coupling appeared markedly lower in SCZ patients, most notably for Pz-frontal and -central electrodes (see CON–SZ difference matrices in Fig. 3d). In contrast to CON, patient SW–spindle coupling appeared insensitive to learning, remaining very similar across baseline and post-learning nights.

### Measures of SW dynamics predict memory consolidation more accurately in controls than patients

To establish whether variables that describe SW, spindles and their coupling predict sleep-dependent changes in MST performance, we built regression models for each variable set and group, enabling unbiased detection of which EEG features best predict behavioural change. A given variable set contained values of that measure for all electrodes/pairs; we then regressed NREM sleep measures during the learning night for each variable set (e.g. SW coherence) against the percentage of overnight improvement in MST (based on the number of correct sequences).

Partial least-squares regression (PLSR) is well-suited to this task; it is similar to principal component regression and is very tolerant of high collinearity in predictor variables.^57,58^ The prediction error of overnight MST improvement from each model reflects the explanatory power of that variable, serving here to quantify the contributions of SW coherence and SW–spindle PAC to sleep-dependent memory consolidation of MST performance. The larger the prediction error, the less accurately that variable predicts behavioural change; smaller residual sum of squares (RESS) values equate to better prediction, with RESS = 0 meaning perfect prediction. Thus, a difference in prediction error between patients and controls may indicate a functional NREM deficit in patients. Models for NREM event properties, SW and spindles and other variables quantifying the long-range interactions of SW and spindles (e.g. SW-associated spindle power and SW-associated spindle coherence) are presented in the Supplementary Information (Supplementary Table S4).

**Fig. 4:**
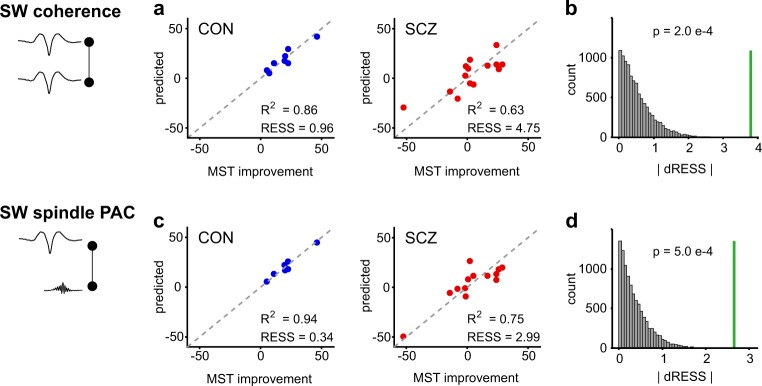
SW coherence and SW–spindle PAC predict sleep-dependent memory consolidation more accurately in CON than in SCZ. **a** PLSR prediction of MST improvement from SW-triggered slow coherence. The PLSR models show that SW coherence is a better predictor (i.e. shows smaller prediction error) of overnight MST change in CON (blue symbols, residual sum of squares, RESS = 0.96, R^2^ = 0.86 using 3 components) compared with SCZ (red symbols, RESS = 4.75, R^2^ = 0.63). **b** Permutation test result for |dRESS_CON–SCZ_| by using SW-triggered slow coherence as a predictor. The absolute sample difference in RESS (|dRESS| = 3.79, green line) is significantly different between CON and SCZ groups (*p* = 2.0e–4 permutation test, *n* = 10,000, see also Supplementary Table S4, alpha = 0.0033). **c** PLSR prediction of MST improvement from SW–spindle PAC. The PLSR model for SW–spindle PAC shows the smallest prediction error for MST change in CON (blue symbols, RESS = 0.34, R^2^ = 0.94), but significantly larger prediction error for MST change in SCZ (red symbols, RESS = 2.99, R^2^ = 0.75). **d** Permutation test result for |dRESS_CON–SCZ_| by using SW–spindle PAC as a predictor. The absolute sample difference in RESS (|dRESS| = 2.65, green line) is significantly different between CON and SCZ groups (*p* = 5.0e–4 permutation test, *n* = 10,000, see also Supplementary Table S4, alpha = 0.0033)

Figure 4 shows the final prediction error results for SW coherence and SW–spindle PAC PLSR components. SW coherence is a better predictor of MST improvement in controls compared with patients (CON: RESS = 0.96, R^2^ = 0.86, SCZ: RESS = 4.75, R^2^ = 0.63). Long-range SW–spindle PAC is overall the best predictor in controls (Fig. 4c, CON: RESS = 0.34, R^2^ = 0.94) compared with all other NREM sleep variables (Supplementary Table S4). SW–spindle PAC in patients is a worse predictor of percentage improvement than that in controls (SCZ: RESS = 2.99, R^2^ = 0.75). Permutation tests with 10,000 permutations of group memberships indicate that group differences in RESS are significant (SW coherence, RESS_CON–SCZ_, *p* = 2.0e–4, SW PAC, RESS_CON–SCZ_, *p* = 5.0e–4) at the Bonferroni-corrected alpha level of 3.3e–3 (Fig. 4b, d and Supplementary Table S4).

This PLSR analysis confirms and extends the results of linear mixed-model analyses of individual electrode pair slow coherence and SW–spindle PAC, that show specific learning-dependent increases in both measures only in the control group (Figs 2 and 3). In conclusion, the extents of SW–SW and SW–spindle coupling are accurate predictors of sleep-dependent memory consolidation in healthy controls, but less predictive in patients.

## Discussion

We analyzed NREM sleep EEG data from patients diagnosed with SCZ and healthy controls, translating predictions arising from a rat model of impaired neurodevelopment that showed dyscoordination of NREM slow waves and spindles across the cortical mantle.^43^ We found striking similarities between patient and rodent EEG: impaired long-range SW coordination, in particular between frontal and occipital cortices, and a disrupted nesting of centro-parietal spindles in frontal SW. These impairments in patients were most prominent following training in a motor sequence task since NREM EEG in SCZ patients failed to show the experience-dependent increases in coordination evident in controls. Quantifying the network coordination of SW and spindles during NREM therefore generates objective measures that predict sleep-dependent memory consolidation in healthy participants, and impaired sleep-dependent memory in SCZ patients.

There is mixed evidence for SW/delta deficits in patients with SCZ: a meta-analysis^59^ failed to confirm reports of altered spectral power in the SW/delta range,^19,21^ but recent studies do link reduced delta power and K-complex density in patients to cognitive deficits^17,25^ and suggest that SW abnormalities are not solely downstream of medication, since they are evident in first-episode psychosis patients.^60^ Yet, our own spectral analysis shows increased low-frequency power during N2 and N3 sleep in our patient sample (see Supplementary Fig. S1). These variable findings likely reflect a multitude of factors, including EEG frequency band definitions, analysis procedures, averaging power measures over multiple sleep stages, medication and behavioural context.

To enhance sensitivity in this study, we focused selectively on spindle and SW-rich sleep stages N2 and N3, anchoring analyses to individually detected SW events. By confining spectral measures of SW amplitude and coherence to time windows surrounding these SW events, we minimised contamination by slow oscillatory noise. This approach revealed a significant reduction of frontal–occipital SW coherence in SCZ during both N2 and N3 sleep. This is the first report of disrupted experience-dependent SW coordination in SCZ, and although progressive habituation to the sleep lab could, in principle, contribute to SW changes across nights, it is reminiscent of the loss of frontal–occipital SW coherence during NREM sleep in the MAM–E17 rat model.^43^ Note that this reduced SW coherence is very unlikely to be secondary to changes in SW occurrence or detection since SW densities and amplitudes were conserved in SCZ patients.

SW coherence has previously been shown to increase after learning a declarative, word-pair task,^50^ and phase synchrony of SW oscillations between motor and sensory cortices has been shown to be necessary and sufficient for successful sleep-dependent consolidation of perceptual learning in rodents.^52^ This supports a model whereby synchronised SW transitions shape distributed ensemble reactivations thought to underlie sleep-dependent memory consolidation; this coordinated activity is evidently impaired in patients with SCZ.

The UP–DOWN state transitions of cortical pyramidal neurons that underpin SW in EEG are disrupted in the MAM–E17 model, potentially reflecting a failure to temporally integrate convergent synaptic inputs^42^ that may contribute to SW abnormalities in SCZ. However, SW coordination also relies on thalamocortical circuits^47^ that are compromised in SCZ.^61,62^ Whether the SW phenotype we report here derives from cortical, thalamocortical or corticothalamic dysfunction therefore remains an important open question.

There is also previous evidence for disrupted SW–spindle interactions in SCZ. A recent analysis of this same dataset showed that the consistency of “local” SW–spindle coupling—i.e. on the same sensor—was predictive of MST memory in SCZ patients, but not in controls.^44^ By extending these analyses to cortex-wide SW–spindle interactions, we demonstrate a learning-dependent increase in fronto-parietal/occipital slow-wave phase to spindle-amplitude coupling in healthy controls, but not in patients. Thus, while local processing during NREM sleep^5,63,64^ clearly supports aspects of sleep-dependent memory consolidation,^65^ deficits in patients with SCZ may derive from compromised top–down modulation of parietal and occipital spindle-associated activity during frontally generated SW.

The learning-specific increase in fronto-occipital SW–spindle coupling is confirmed by regression analysis, showing that brain-wide SW–spindle coupling is overall the best predictor of sleep-dependent memory consolidation in healthy controls, with coupling involving parietal regions particularly prominent. A higher prediction error in patients suggests a deficit in functionally relevant SW–spindle coupling.

The precise mechanisms underlying SW-phase to spindle-amplitude coupling remain unclear, but given the considerable overlap of circuits involved in the generation of SW and spindles,^47,66–68^ circuit defects in schizophrenia may affect the generation and coordination of both NREM oscillations in patients.^39^ Dysfunctional GABAergic transmission may be central to this SCZ phenotype; for example, the GABA receptor agonist zolpidem has been shown to enhance SW–spindle coupling and memory in healthy participants.^69^

The succession of SW and subsequent spindle event may not only signify local processing. We show that SW synchronisation between remote cortical areas coincides with long-range SW-phase to spindle-amplitude coupling, potentially enabling coordinated information exchange between distant cortical networks and thus facilitating overnight procedural memory consolidation. SW-triggered processing can also affect the hippocampus that has been shown to be involved in some types of motor sequence learning.^70–73^ In both rats and humans, it has been shown that SW influences the timing of spindles, which are in turn synchronised to fast hippocampal ripple oscillations.^35–37,74,75^ This intricate synchronisation of sleep oscillations might represent a mechanism for targeted information integration from the associational networks in the hippocampus during encoding to long-term storage sites in the cortex.^76^

This precise temporal organisation is impaired in patients with SCZ; hence, oscillatory sleep events constitute biomarkers of circuit dysfunction.^39^ In particular, using SW as markers for detailed analyses of spectral dynamics allows focusing on key time windows of thalamocortical activity and function during NREM sleep, minimising variance introduced by attention or other task variables present during wake behaviour. SW and other oscillatory events during sleep provide an internal standard and a data-driven approach to compare patient and healthy control data and should also frame the comparison of animal and human data in future translational studies.

Our findings add to a growing body of evidence that sleep should be routinely considered in relation to cognitive deficits in neuropsychiatry. Current therapies have very limited positive impact on cognition in patients, but future clinical and research strategies should account for the potential of medications to disrupt or augment sleep-dependent network activity. In the future, high-resolution sleep neurophysiology and analyses of network dynamics have the potential to improve diagnosis and target treatments on an individual patient basis, enabling a neurobiologically informed stratification that has, to date, proved so elusive.

## Methods

### Participants

Here we analyzed a previously published dataset presented in ref. ^29^ Briefly, 21 schizophrenia outpatients were recruited from an urban mental health centre. Diagnoses were confirmed with Structured Clinical Interviews for DSM-IV and symptoms rated according to the Positive and Negative Syndrome Scale.^77,78^ The control group of 17 healthy participants were screened to exclude a personal history of mental illness, family history of schizophrenia spectrum disorder and psychoactive medication use. Some datasets in both the CON group (baseline and learning sleep) and the SCZ group (only baseline sleep) were excluded from further analysis due to high low-frequency noise levels that affected the reliable detection of slow waves (see details of missing data and channels in Supplementary Information, Tables S1–S3).

The remaining patient (night 1, *n* = 17, night 2, *n* = 21) and control (night 1 = *n* = 15, night 2, *n* = 15) participants did not differ in age, sex or parental education. All participants were screened for diagnosed sleep disorders, treatment with sleep medications, a history of head injury, neurological illness and substance abuse or dependency. All participants gave written informed consent. All experiments were performed according to the International Conference on Harmonization Clinical Good Practice guidelines. The study was approved by the Institutional Review Boards of Massachusetts General Hospital, the Massachusetts Department of Mental Health and Beth Israel Deaconess Medical Center.

### Sleep recordings and behaviour

Participants visited the Clinical Research Center (CRC) the week before their stay to complete informed consent, demographic questionnaires and rating scales. They also received an actigraph to wear until study completion (see ref. ^29^ for full Methods and Results).

EEG and polysomnography (PSG) data were recorded during 2 consecutive weeknights in the CRC with participants engaging in their usual activities during the intervening day. On the second night, participants were trained on the motor sequence task (MST) 1 h before their usual bedtime, wired for PSG and allowed to sleep for up to 10 h. They were tested on the MST again 1 h after awakening.

### Motor sequence task

The MST requires pressing four numerically labelled keys in a five-element sequence (4-1-3-2-4) on a standard computer keyboard with the fingers of the left hand. The numeric sequence was displayed on a computer screen, with dots under each number that indicated a keystroke. Sequences have to be entered “as quickly and accurately as possible” over 30-s trials.

During both training and test sessions, participants had to alternate between typing and resting for 30 s for a total of 12 trials, with the number of correct sequences per trial reflecting the speed and accuracy of performance. Overnight improvement was calculated as the percent increase in correct sequences from the last three training trials to the first three test trials the following morning (Fig. 1c).

### Polysomnography

Different montages of five to eight channels (F3, F4, C3, Cz, C4, Pz, O1 and O2) were placed according to the standard 10–20 system, and EEG data digitised at 100 Hz by using an Embla N7000 system (Medcare Systems, Buffalo, New York). EEG was referenced to the linked mastoids for further analysis. Recordings were divided into 30-s epochs and scored according to standard criteria^79^ as WAKE, REM, N1, N2 and N3 sleep by expert scorers blind to recording night and diagnosis. Thirty-second epochs with high noise levels were manually discarded.

### SW and spindle detection

Sleep EEG oscillatory events were detected as described in ref. ^43^ SWs were detected from 0.25- to 4-Hz bandpass-filtered EEG: the whole EEG trace (noisy epochs removed) was converted into a z score, and threshold crossings above 3.5 standard deviations (SD) from mean amplitude were detected as candidate events. Here we have been purposefully agnostic to the traditional K-complex and delta wave definitions that rely on the occurrence of events in different sleep stages and other morphological parameters. We (and others) believe that the underlying biophysical mechanisms for SW, K complexes and delta waves are likely to be closely related.^80–82^ Candidate events were only accepted as SW if they fell within the following parameter ranges: amplitude 50–300 µV; length 0.2–3 s; minimum gap between SW to be considered separate events 0.5 s (Supplementary Fig. S2a). All analyses presented here are based on negative singular threshold crossings with a frequency (period of oscillation) below 1.5 Hz.

To detect spindles, EEG traces were bandpass filtered (9–16 Hz), z-scored and rectified, and an envelope of the rectified signal was determined by using a cubic spline fit to the maxima of the rectified signal. Candidate spindle events were detected as threshold crossings of above 3.5 SD of the envelope, then classified as spindles given: amplitude 25–500 µV; length 0.25–3 s; minimum gap between spindles to be considered separate events 0.25 s; start/end limit threshold 1.5 SD; oscillation frequency 12–15 Hz. Differences between SW amplitudes, SW–spindle cross-correlation bins and spectrogram bins were assessed by using a 2-tailed Wilcoxon rank-sum test with subsequent FDR correction^83^ for multiple testing.

### EEG spectral analyses

The average spectra for stage 2 sleep and event-triggered spectral analyses were derived by using multi-tapered spectra/spectrograms and coherograms by using the Chronux toolbox^41^ (www.chronux.org). SW negative peak times were used as triggers to analyze 4-s windows of EEG data (±2 s around each SW). The event-triggered spectrograms and coherograms were then calculated by using 3 tapers, a 1-s sliding-data window with 50-ms steps and a bandwidth of 0.1 (for SW) or 1 Hz (spindle analyses) and subsequently averaged.

### Phase-amplitude coupling

SW event-centred EEG windows were also used to calculate phase-amplitude coupling (PAC) by using a set of previously described custom MATLAB routines.^56^ We used the modulation index (MI) to quantify low-frequency oscillation (0.5–5 Hz) phase to fast oscillation- (5–20 Hz) amplitude coupling. The MI can quantify phase-amplitude coupling within a signal or between signals from different sensors. Briefly, the modulation index is calculated as follows: both the slow-frequency signal and the fast-frequency signal (which can be identical if we look for PAC in the same signal, i.e. local PAC) are bandpass filtered at their respective frequency of interest. Next, the phase of the slow-frequency signal and the amplitude of the fast-frequency signal are calculated by using the Hilbert transform, respectively. This results in a phase and an amplitude time series used to create a histogram of amplitude values per phase value. If the phase of the slower-frequency signal had no influence on the amplitude of the faster-frequency signal, we would expect a uniform distribution of amplitude values across phase bins. The modulation index thus quantifies the divergence of the amplitude distribution from a uniform distribution by using a modified Kullback–Leibler distance^55,84^ where an MI of 0 indicates no phase-amplitude coupling and an MI of 1 would indicate a Dirac-like distribution with all amplitude values appearing in one phase bin. We initially detected a modulation of slow-wave phase (0.5–1.5 Hz) to fast spindle oscillation (12–15 Hz) amplitude by using a wider frequency range (Fig. 3) and subsequently ran MI calculation for SW–spindle PAC on all possible electrode pairs to quantify slow-wave to fast-spindle brain-wide cross-frequency coupling during NREM sleep.

Some analysis routines were used in combination with the Matlab^©^ Parallel Computing Toolbox, and all calculations were run on a Dell Precision Tower 7810 with two Intel^®^ Xeon^®^ CPU E5-2667 at 3.20 GHz and 32 GB of RAM.

### General statistical methods

We used two-sided *t* tests to analyze differences in behavioural variables, and the Wilcoxon rank-sum test with FDR correction to test bin-wise differences in spectral power between groups.

### Linear mixed-model analysis

To analyze the changes in coherence or PAC for selected electrode pairs we used a linear mixed-model approach.^85^ To test for the main and interaction effects of group and recording night, event-triggered coherence and MI values for both nights for groups CON and SCZ were entered into random intercept models, where variability between subjects is accounted for by modelling the participant ID as a random effect (i.e. allowing individuals to have their own intercept about the group mean), and night (baseline vs learning) and group (CON vs SCZ) are added as fixed effects. Models were implemented by using the “lme4” library^86^ in the R environment.^87^ Interaction terms were included if they improved the model significantly (as assessed by a model comparison by using a likelihood ratio test using maximum likelihood estimated versions of the models). If interaction terms were added to the final model, least-squares estimation of group means and differences for each night and post hoc multiple comparisons were calculated by using the “lmerTest” library^88^ where p values were calculated from F statistics of type III by using Satterthwaite approximation of degrees of freedom.

### Data preparation and PLS regression model building

Sleep predictor variable sets to enter regression models were gathered into a wide table data format and were chosen to be average detected NREM event properties (SW and spindles) or connectivity measures such as SW coherence, SW–spindle PAC (and for comparison SW-triggered spindle coherence and spindle-triggered spindle coherence). We only considered measures of NREM sleep during learning night 2. The MST test results were entered as the percent change in the number of correct sequences from the last 3 trials in MST1 to the first 3 trials in MST2 (Fig. 1). NREM sleep electrophysiology variables and change in MST were transformed to z-scores (across both groups), allowing comparisons of model performance in multiples of the standard deviation for the whole population. Complete cases were then extracted for each group and modelled separately (with different levels of missingness). We compared model performances with reduced sets of variables or group sizes, and the results were comparable to the final models presented here.

Since the number of NREM sleep electrophysiology variables was greater than the number of individuals (p < N), partial least- squares (PLS) regression^57,58,89^ was used to reduce information in all variable sets into a smaller number of principal components (PC). PLS regression is similar to principal component regression in terms of dimensionality reduction, but in PLS regression, the outcome (Y) is included in the data reduction step. PLS regression works by reducing the exposure variables into PCs, which have the greatest covariance with the outcome (Y). Therefore, the resulting PCs represent relevant structural information about the outcome Y (Supplementary Statistical Methods SM6).

PLS regression was carried out separately on each of the sleep exposure sets (connectivity variables) and each of the two groups (Supplementary Table S4). In order to compare prediction performance of the sleep exposure sets, it was decided a priori to choose 3-component models, where the explained outcome variance in the model for the EEG-derived connectivity predictor X had to reach at least 80% of the total variance at least in one of the groups.^90^ PLS models were also built with 6 components to confirm the stability of the presented results (not shown). For comparison, we also present all model results for non-connectivity-based sleep exposures (NREM event properties and power measures, Supplementary Table S4) although we relaxed the 80% explained variance rule for these variable sets. The outcome (y) was taken to be the percentage overnight change in MST performance as described above. PLS regression was carried out in Matlab (Mathworks, Natick, MA) by using the built-in function *plsregress*. The residual sum of squares (RESSS) and R^2^ were used to compare models between groups and exposure sets.^89^

We used permutation tests to assess whether the difference in RESS values between groups is likely to be significant. We computed 10,000 permutations of group membership (CON, SCZ) with fixed group sizes to estimate the null distribution, i.e. no difference in RESS between randomly composed groups (dRESS = 0). We then computed a PLSR model for each of the permutations and each variable set to compare the sample difference in RESS with the null distribution. Two-sided *p* values were calculated as the number of absolute dRESS values that are bigger than the sample value divided by the total number of permutations. The final *p* values were corrected by using a Bonferroni threshold of 0.005/15 = 0.0033, given that we tested 15 sleep variable sets.

### Reporting summary

Further information on research design is available in the Nature Research Reporting Summary linked to this article.

## Supplementary information

Supplementary Information

Reporting summary

## Data Availability

Data will be made available on request.

## Code availability

All custom code will be made available on request. An updated version of the NREM event (SW and spindle) detection algorithm is available at https://gitlab.com/ubartsch/sleepwalker.
